# Pattern of placental alkaline phosphatase (PLAP) expression in human tumors: a tissue microarray study on 12,381 tumors

**DOI:** 10.1002/cjp2.237

**Published:** 2021-08-07

**Authors:** Viktor Reiswich, Natalia Gorbokon, Andreas M Luebke, Eike Burandt, Anne Menz, Martina Kluth, Claudia Hube‐Magg, Corinna Wittmer, Sören Weidemann, Christoph Fraune, Katharina Möller, Patrick Lebok, Guido Sauter, Ronald Simon, Ria Uhlig, Waldemar Wilczak, Frank Jacobsen, Sarah Minner, Rainer Krech, Christian Bernreuther, Andreas Marx, Stefan Steurer, Till Clauditz, Till Krech

**Affiliations:** ^1^ Institute of Pathology University Medical Center Hamburg‐Eppendorf Hamburg Germany; ^2^ Institute of Pathology Clinical Center Osnabrueck Osnabrueck Germany; ^3^ Department of Pathology Academic Hospital Fuerth Fuerth Germany

**Keywords:** immunohistochemistry, PLAP, tissue microarray

## Abstract

Placental alkaline phosphatase (PLAP) is commonly expressed at high levels in testicular germ cell tumors. PLAP immunohistochemistry (IHC) is thus often used to confirm this diagnosis, especially in cases of putative metastasis. However, other tumors can also express PLAP. To comprehensively determine PLAP expression in normal and tumor tissue, a tissue microarray containing 16,166 samples from 131 different tumor types and subtypes as well as 608 samples from 76 different normal tissue types was analyzed by IHC. Moderate to strong PLAP positivity was found in 27 (21%) of 131 different tumor types including seminoma (96%), embryonal carcinoma (85%), and yolk sac tumors of the testis (56%); endometrioid carcinoma of the endometrium (28%) and the ovary (20%); gastric adenocarcinoma (22%); serous carcinoma (not otherwise specified) of the ovary (17%) and the uterus (11%); adenocarcinoma of the ampulla of Vater (15%); carcinosarcoma of the ovary (11%) and the uterus (8%); esophageal adenocarcinoma (10%); invasive urothelial carcinoma (4%); cholangiocarcinoma (2%); and adenocarcinoma of the lung (1%). Low‐level PLAP immunostaining, often involving only a small fraction of tumor cells, was seen in 21 additional tumor entities. The clinical significance of PLAP expression may vary between tumor types as high PLAP expression was linked to advanced pathological tumor stage (*p* = 0.0086), nodal metastasis (*p* = 0.0085), and lymphatic (*p* = 0.0007) and blood vessel invasion (*p* = 0.0222) in colorectal cancer, but to low pathological tumor stage in endometrial cancer (*p* = 0.0043). In conclusion, our data identify several tumor entities that can show PLAP expression at comparable levels to testicular germ cell tumors. These tumor entities need to be considered in cases of PLAP‐positive metastasis. Low‐level PLAP expression can be found in various other tumor entities and should generally not be viewed as a strong argument for germ cell neoplasia.

## Introduction

Placental alkaline phosphatase (PLAP), also known as alkaline phosphatase, placental type (ALPP), is encoded by the *ALPP* gene at chromosome 2q37.1 [1]. PLAP is a dimer of 65 kDa consisting of 535 amino acids and is thought to play a role in guiding migratory cells and transport specific molecules over the plasma membrane [1, 2]. PLAP is expressed in the placenta from the ninth week of gestation and its concentration increases continually throughout pregnancy [2]. PLAP can be separated into three distinct isoenzymes corresponding to early, mid, and term placenta [3]. In normal human tissues, the expression of PLAP is largely restricted to the placenta but low‐level RNA expression has also been reported for uterine cervix, fallopian tube, and – to a lower level – the lung [4, 5].

PLAP expression also occurs in tumors [4, 6, 7, 8, 9, 10, 11, 12, 13, 14, 15, 16, 17, 18, 19, 20, 21, 22, 23, 24, 25, 26, 27, 28, 29, 30, 31, 32, 33, 34, 35, 36, 37, 38, 39, 40, 41, 42, 43, 44, 45, 46, 47, 48, 49, 50, 51, 52, 53, 54, 55, 56, 57, 58, 59, 60]. This is particularly known for testicular germ cell tumors [6, 7, 8, 10, 11, 12, 13, 14, 15, 16, 17, 18, 19, 20, 21, 22, 23, 24, 25, 26, 27, 28, 29, 30, 31, 32, 33, 34, 35, 36]. PLAP expression, often at high levels, has been described to occur in up to 100% of testicular germ cell neoplasia *in situ* [20, 28, 30], up to 100% of seminoma [4, 6, 7, 9, 20, 21, 22, 23, 30, 35, 36, 38, 41, 42], up to 100% of embryonal carcinoma [35, 42], up to 87% of yolk sac tumors [30], and up to 100% of choriocarcinomas [12, 16, 19]. Antibodies targeting PLAP are thus regularly used for the detection and classification of testicular tumors [61]. Various studies have demonstrated, however, that PLAP expression can also occur in non‐germinal cell tumors, but immunohistochemical data are controversial. For example, positive PLAP immunostaining has been described to occur in 0–100% of adenocarcinomas of the ampulla of Vater [4, 35], 0–38% of gastric adenocarcinoma [4, 35], 0–100% of rhabdomyosarcomas [44, 56], 20–80% of high‐grade serous carcinomas of the ovary [43, 59], and 0–18% of clear cell renal cell carcinomas [4, 35]. These conflicting data are probably caused by the use of different antibodies, immunostaining protocols, and criteria to determine PLAP positivity in these studies.

To better understand the prevalence of PLAP immunostaining in different tumor types, a comprehensive study analyzing a large number of neoplastic and non‐neoplastic tissues under highly standardized conditions is needed. We thus analyzed PLAP expression in more than 16,000 tumor tissue samples from 131 different tumor types and subtypes as well as 76 non‐neoplastic tissue types by immunohistochemistry (IHC) in a tissue microarray (TMA) format.

## Materials and methods

### Tissue microarrays

TMAs composed of normal and tumor tissues were employed for this study. The normal TMA contained eight samples from eight different donors from each of 76 different normal tissue types. The cancer TMAs contained a total of 16,166 primary tumors from 131 tumor types and subtypes. Histopathological data including grade, pathological tumor (pT) stage, and pathological lymph node (pN) status were available from 583 ovarian cancers, 259 endometrial cancers, and 1,784 colorectal cancers. The dataset on colorectal cancer also included molecular information on mismatch repair protein deficiency. The composition of both normal and cancer TMAs is described in the Results section. All samples were from the archives of the Institutes of Pathology, University Hospital of Hamburg, Germany; the Institute of Pathology, Clinical Center Osnabrueck, Germany; and Department of Pathology, Academic Hospital Fuerth, Germany. Tissues were fixed in 4% buffered formalin and then embedded in paraffin. TMA tissue spot diameter was 0.6 mm. The use of archived remnants of diagnostic tissues for manufacturing of TMAs and their analysis for research purposes as well as patient data analysis has been approved by local laws (HmbKHG, §12) and by the local ethics committee (Ethics commission Hamburg, WF‐049/09). All work was carried out in compliance with the Helsinki Declaration.

### Immunohistochemistry

Freshly cut TMA sections were immunostained under the same experimental conditions. Two different primary antibodies were used for PLAP detection: MSVA‐350R (rabbit recombinant; MS Validated Antibodies, Hamburg, Germany) and IR779 (mouse monoclonal 8A9, Agilent DAKO, Santa Clara, CA, USA). The normal tissue array was analyzed with both MSVA‐350R and IR779, while the multitumor array was analyzed with MSVA‐350R only. For MSVA‐350R, slides were deparaffinized with xylol, rehydrated through a graded alcohol series, and exposed to heat‐induced antigen retrieval for 5 min in an autoclave at 121°C in pH 9.0 Target Retrieval Solution (Agilent). Endogenous peroxidase activity was blocked with Peroxidase Blocking Solution (Agilent) for 10 min. The primary antibody was diluted 1:150 and applied for 60 min at 37°C. For IR779, the slides were deparaffinized and rehydrated as described previously, and exposed to heat‐induced antigen retrieval for 15 min in Agilent's PT Link pretreatment module at 95°C in pH 9.0 retrieval buffer. Slides were transferred to an Autostainer Link 48 device (Agilent) for peroxidase blocking (5 min) and incubation of the primary antibody (ready to use prediluted for 20 min at room temperature). Both antibodies were visualized using the respective EnVision reagents (Agilent) for manual and automated staining according to the manufacturer's directions. One pathologist (NG) analyzed all immunostainings. For tumor tissues, the percentage of positive neoplastic cells was estimated, and the staining intensity was semiquantitatively recorded (0, 1+, 2+, and 3+). For statistical analyses, the staining results were categorized into four groups. Tumors without any staining were considered negative. Tumors with 1+ staining intensity in ≤70% of cells and 2+ intensity in ≤30% of cells were considered weakly positive. Tumors with 1+ staining intensity in >70% of cells, 2+ intensity in 31–70%, or 3+ intensity in ≤30% were considered moderately positive. Tumors with 2+ intensity in >70% or 3+ intensity in >30% of cells were considered strongly positive.

### Statistics

Statistical calculations were performed with JMP 14 software (SAS Institute Inc., Cary, NC, USA). Contingency tables and the chi‐square test were performed to search for associations between PLAP and tumor phenotype. Survival curves were calculated according to Kaplan–Meier. The log‐rank test was applied to detect significant differences between groups. A *P* value of ≥0.05 was considered as statistically significant.

## Results

### Technical issues

A total of 12,381 (76.6%) of 16,166 tumor samples were interpretable in our TMA analysis. Non‐interpretable samples demonstrated lack of unequivocal tumor cells or loss of the tissue spot during technical procedures. A sufficient number of samples of each normal tissue type was evaluable.

### PLAP in normal tissues

**Figure 1:**
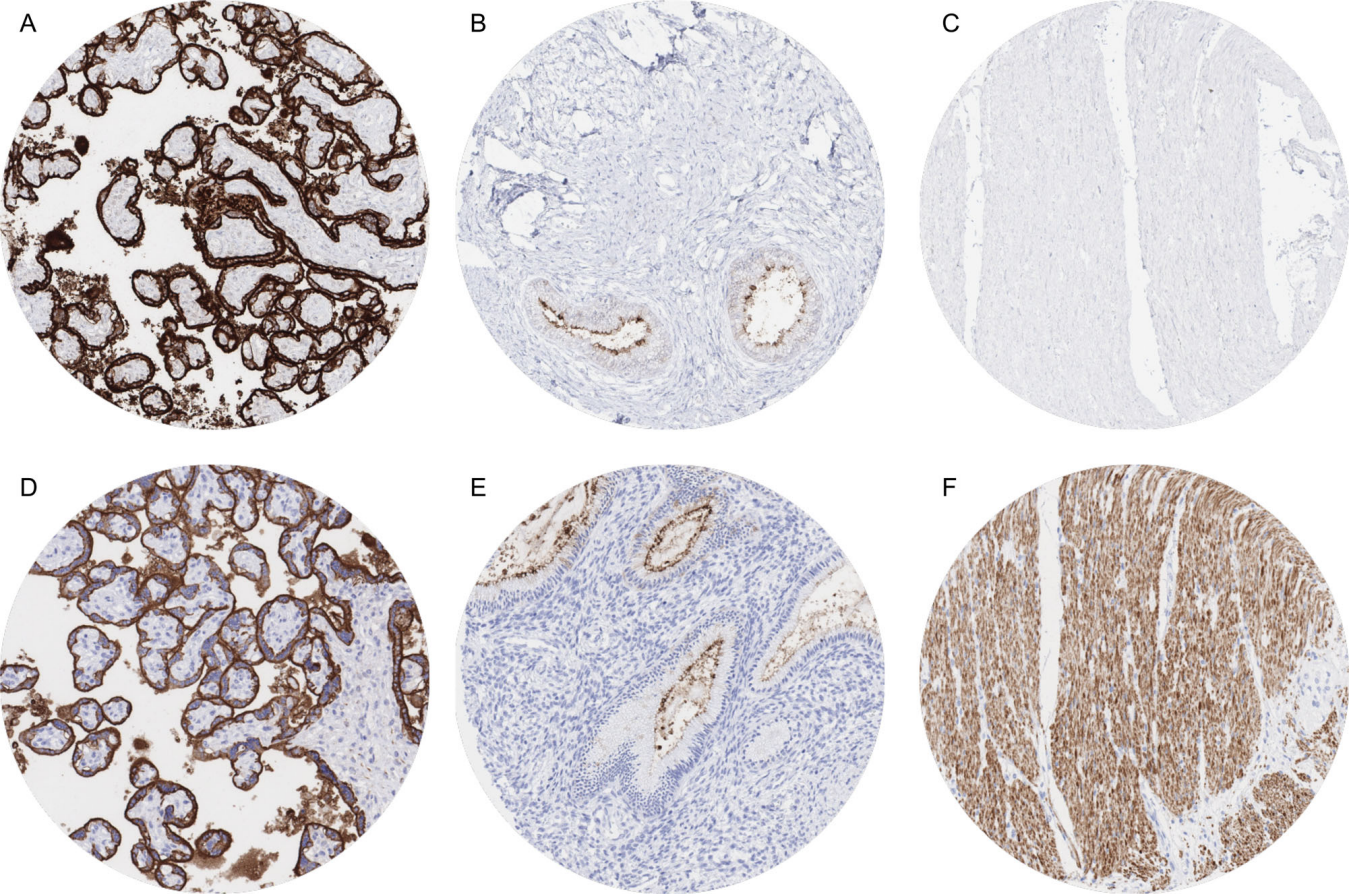
PLAP immunostaining of normal tissues (comparison of antibodies). The panels show (A) strong PLAP positivity of trophoblastic cells in the placenta, (B) weak to moderate apical staining of endocervical glands, and (C) absence of staining in smooth muscle from the colon wall for the antibody MSVA‐350R. Using the antibody IR779, identical findings are seen for (D) placenta and (E) endocervical glands but an additional strong staining occurred in (F) smooth muscle cells.

With two different antibodies (MSVA‐350R and IR779), particularly strong PLAP immunostaining was found in the placenta. Here, strong PLAP positivity was seen in chorion cells as well as in cyto‐ and syncytiotrophoblast of mature placenta (Figure 1A,D). Staining was only moderate and limited to the surface cell membrane in the trophoblast of early placenta, and only weak in amnion cells. Also, for both antibodies, weak PLAP staining was seen at the apical membrane of epithelial cells in the endocervix (Figure 1B,E), endometrium, and the fallopian tube, although this did not occur in all samples analyzed. PLAP immunostaining was lacking for both antibodies in most other tissues including all epithelial cells of the gastrointestinal and the genitourinary tract, gallbladder, liver, pancreas, salivary and bronchial glands, breast glands, Brunner glands, thyroid, pituitary gland, adrenal gland, parathyroid gland, testis, epididymis, seminal vesicle, prostate, non‐keratinizing and keratinizing squamous epithelium of various different sites, skin appendages, hematopoietic and immune cells, and the brain. Staining of muscular tissues revealed complete absence of staining by MSVA‐350R (Figure 1C) while Agilent Dako IR779 showed moderate to strong staining of smooth muscle (Figure 1F) and weak to moderate staining of skeletal muscle. These latter findings were considered to be due to cross‐reactivity.

### PLAP in cancer

**Figure 2:**
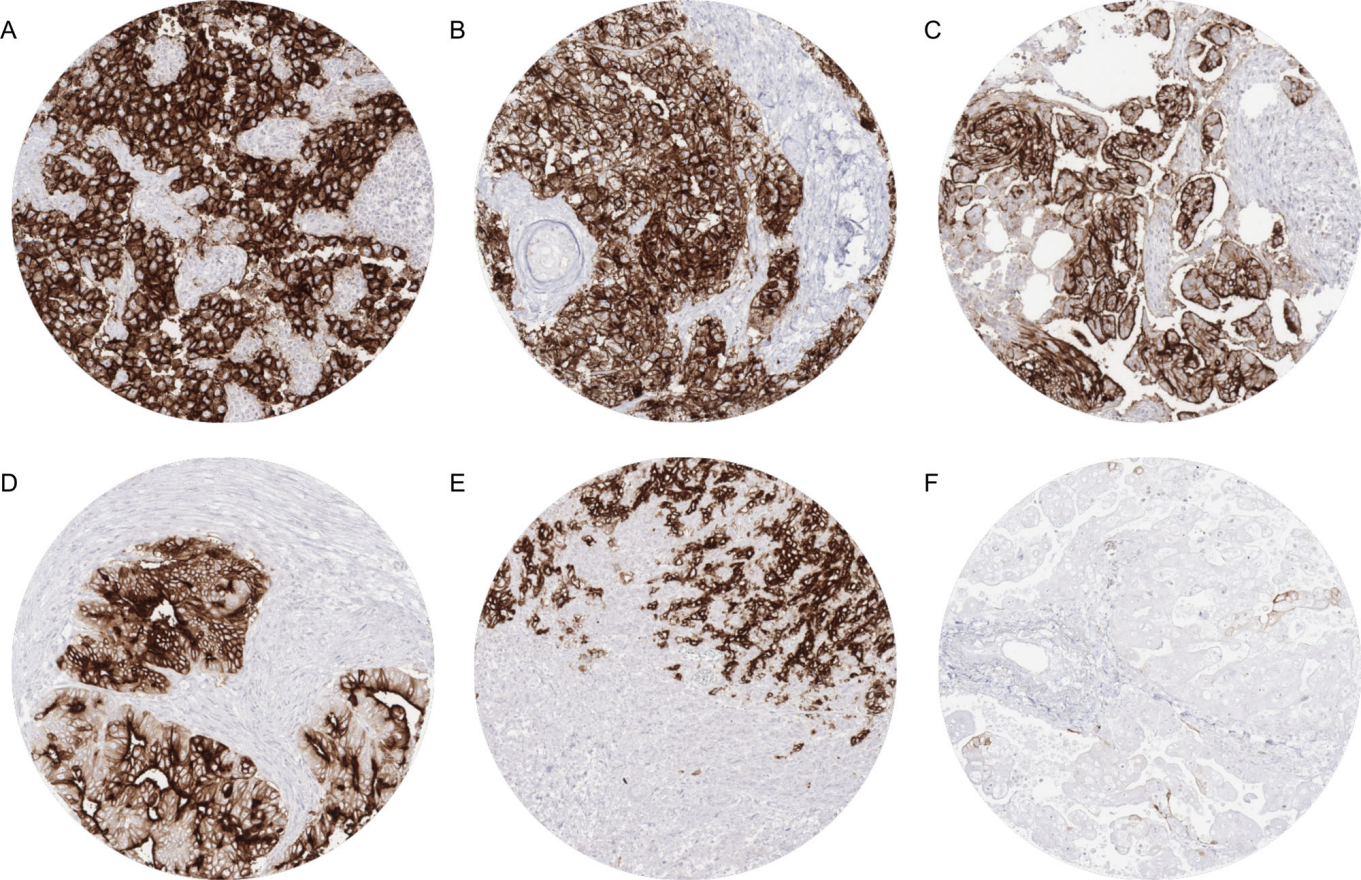
PLAP immunostaining in cancer. The panels show strong PLAP positivity in (A) seminoma, (B) embryonal carcinoma, (C) high‐grade serous carcinoma of the ovary, (D) adenocarcinoma of the pancreas, and (E) gastric adenocarcinoma, and weak focal PLAP positivity in (F) an adenocarcinoma of the lung with the antibody MSVA‐350R.

**Figure 3:**
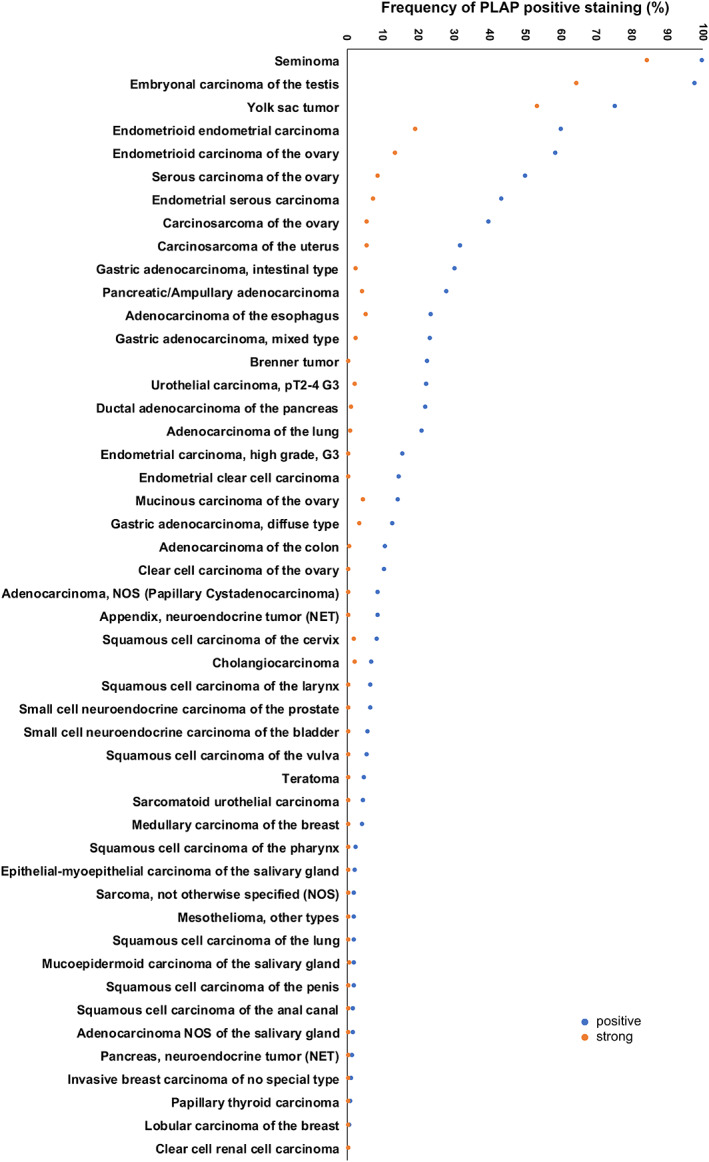
Ranking order of PLAP immunostaining in human tumors. Both the frequency of positive cases (blue dots) and the frequency of strongly positive cases (orange dots) are shown. Eighty‐three additional tumor entities without any PLAP‐positive cases are not shown due to space restrictions.

By using MSVA‐350R, positive PLAP immunostaining was detectable in 1,503 (12.1%) of the 12,381 analyzable tumors, including 761 (6.1%) with weak, 184 (1.5%) with moderate, and 558 (4.5%) with strong immunostaining. Overall, 48 (36.6%) of 131 tumor categories showed detectable PLAP expression with 22 (16.8%) tumor categories showing strong positivity in at least one case (Table 1). Representative images of PLAP‐positive tumors are shown in Figure 2. The highest rate of positive staining was found in testicular tumors, followed by tumors of the female genital tract, gastroesophageal, and pancreaticobiliary cancers. It is of note that only weak PLAP immunostaining was occasionally found in 21 different tumor entities. In most of these tumors, PLAP immunostaining was limited to a small fraction of tumor cells (Figure 2D,E). None of the 48 leiomyomas, 84 leiomyosarcomas, 7 rhabdomyosarcomas, or 91 angiomyolipomas showed any PLAP staining. A graphical representation of the rank order of PLAP positive and strongly positive cancers is shown in Figure 3.

**Table 1:** PLAP immunostaining in human tumors.

		PLAP immunostaining						
	Tumor entity	On TMA (*n*)	Analyzable (*n*)	Negative (%)	Weak (%)	Moderate (%)	Strong (%)	Positive (%)
**Tumors of the skin**	Pilomatrixoma	35	32	100.0	0.0	0.0	0.0	0.0
	Basal cell carcinoma	88	56	100.0	0.0	0.0	0.0	0.0
	Benign nevus	29	26	100.0	0.0	0.0	0.0	0.0
	Squamous cell carcinoma of the skin	90	82	100.0	0.0	0.0	0.0	0.0
	Malignant melanoma	48	44	100.0	0.0	0.0	0.0	0.0
	Merkel cell carcinoma	46	44	100.0	0.0	0.0	0.0	0.0
**Tumors of the head and neck**	Squamous cell carcinoma of the larynx	110	96	93.8	5.2	1.0	0.0	6.3
	Squamous cell carcinoma of the pharynx	60	46	97.8	2.2	0.0	0.0	2.2
	Oral squamous cell carcinoma (floor of the mouth)	130	118	100.0	0.0	0.0	0.0	0.0
	Pleomorphic adenoma of the parotid gland	50	44	100.0	0.0	0.0	0.0	0.0
	Warthin tumor of the parotid gland	104	96	100.0	0.0	0.0	0.0	0.0
	Adenocarcinoma, NOS (papillary cystadenocarcinoma)	14	12	91.7	8.3	0.0	0.0	8.3
	Salivary duct carcinoma	15	12	100.0	0.0	0.0	0.0	0.0
	Acinic cell carcinoma of the salivary gland	181	143	100.0	0.0	0.0	0.0	0.0
	Adenocarcinoma NOS of the salivary gland	109	79	98.7	1.3	0.0	0.0	1.3
	Adenoid cystic carcinoma of the salivary gland	180	119	100.0	0.0	0.0	0.0	0.0
	Basal cell adenocarcinoma of the salivary gland	25	23	100.0	0.0	0.0	0.0	0.0
	Basal cell adenoma of the salivary gland	101	91	100.0	0.0	0.0	0.0	0.0
	Epithelial–myoepithelial carcinoma of the salivary gland	53	52	98.1	1.9	0.0	0.0	1.9
	Mucoepidermoid carcinoma of the salivary gland	343	262	98.5	1.1	0.0	0.4	1.5
	Myoepithelial carcinoma of the salivary gland	21	20	100.0	0.0	0.0	0.0	0.0
	Myoepithelioma of the salivary gland	11	9	100.0	0.0	0.0	0.0	0.0
	Oncocytic carcinoma of the salivary gland	12	12	100.0	0.0	0.0	0.0	0.0
	Polymorphous adenocarcinoma, low grade, of the salivary gland	41	33	100.0	0.0	0.0	0.0	0.0
	Polymorphous adenoma of the salivary gland	53	35	100.0	0.0	0.0	0.0	0.0
**Tumors of the lung, pleura, and thymus**	Adenocarcinoma of the lung	246	160	79.4	19.4	0.6	0.6	20.6
	Squamous cell carcinoma of the lung	130	65	98.5	1.5	0.0	0.0	1.5
	Small cell carcinoma of the lung	20	16	100.0	0.0	0.0	0.0	0.0
	Mesothelioma, epithelioid	39	32	100.0	0.0	0.0	0.0	0.0
	Mesothelioma, other types	76	63	98.4	1.6	0.0	0.0	1.6
	Thymoma	29	29	100.0	0.0	0.0	0.0	0.0
**Tumors of the female genital tract**	Squamous cell carcinoma of the vagina	78	63	100.0	0.0	0.0	0.0	0.0
	Squamous cell carcinoma of the vulva	130	116	94.8	5.2	0.0	0.0	5.2
	Squamous cell carcinoma of the cervix	130	124	91.9	6.5	0.0	1.6	8.1
	Endometrioid endometrial carcinoma	236	223	40.4	31.8	9.0	18.8	59.6
	Endometrial serous carcinoma	82	72	56.9	31.9	4.2	6.9	43.1
	Carcinosarcoma of the uterus	48	38	68.4	23.7	2.6	5.3	31.6
	Endometrioid carcinoma, high grade, G3	13	13	84.6	15.4	0.0	0.0	15.4
	Endometrial clear cell carcinoma	8	7	85.7	14.3	0.0	0.0	14.3
	Endometrioid carcinoma of the ovary	110	91	41.8	38.5	6.6	13.2	58.2
	Serous carcinoma of the ovary (NOS)	559	462	50.2	32.9	8.4	8.4	49.8
	Mucinous carcinoma of the ovary	96	71	85.9	7.0	2.8	4.2	14.1
	Clear cell carcinoma of the ovary	50	40	90.0	10.0	0.0	0.0	10.0
	Carcinosarcoma of the ovary	47	38	60.5	28.9	5.3	5.3	39.5
	Brenner tumor	9	9	77.8	22.2	0.0	0.0	22.2
**Tumors of the breast**	Invasive breast carcinoma of no special type	1,391	1185	99.2	0.8	0.0	0.0	0.8
	Lobular carcinoma of the breast	294	236	99.6	0.0	0.4	0.0	0.4
	Medullary carcinoma of the breast	26	26	96.2	3.8	0.0	0.0	3.8
	Tubular carcinoma of the breast	27	26	100.0	0.0	0.0	0.0	0.0
	Mucinous carcinoma of the breast	58	44	100.0	0.0	0.0	0.0	0.0
	Phyllodes tumor of the breast	50	47	100.0	0.0	0.0	0.0	0.0
**Tumors of the digestive system**	Adenomatous polyp, dysplasia	1,000	98	100.0	0.0	0.0	0.0	0.0
	Adenocarcinoma of the colon	956	721	89.7	9.3	0.7	0.3	10.3
	Gastric adenocarcinoma, diffuse type	226	130	87.7	8.5	0.8	3.1	12.3
	Gastric adenocarcinoma, intestinal type	224	134	70.1	22.4	5.2	2.2	29.9
	Gastric adenocarcinoma, mixed type	62	48	77.1	18.8	2.1	2.1	22.9
	Adenocarcinoma of the esophagus	133	60	76.7	13.3	5.0	5.0	23.3
	Squamous cell carcinoma of the esophagus	124	42	100.0	0.0	0.0	0.0	0.0
	Squamous cell carcinoma of the anal canal	91	78	98.7	1.3	0.0	0.0	1.3
	Cholangiocarcinoma	114	108	93.5	4.6	0.0	1.9	6.5
	Hepatocellular carcinoma	50	50	100.0	0.0	0.0	0.0	0.0
	Ductal adenocarcinoma of the pancreas	663	459	78.4	19.2	1.5	0.9	21.6
	Pancreatic/ampullary adenocarcinoma	119	76	72.4	13.2	10.5	3.9	27.6
	Acinar cell carcinoma of the pancreas	13	12	100.0	0.0	0.0	0.0	0.0
	Gastrointestinal stromal tumor	50	49	100.0	0.0	0.0	0.0	0.0
**Tumors of the urinary system**	Urothelial carcinoma, pT2‐4 G3	1,207	613	78.0	18.4	1.6	2.0	22.0
	Small cell NEC of the bladder	18	18	94.4	5.6	0.0	0.0	5.6
	Sarcomatoid urothelial carcinoma	25	24	95.8	4.2	0.0	0.0	4.2
	Clear cell renal cell carcinoma	1,226	759	99.9	0.0	0.1	0.0	0.1
	Papillary renal cell carcinoma	320	208	100.0	0.0	0.0	0.0	0.0
	Clear cell (tubulo) papillary renal cell carcinoma	28	19	100.0	0.0	0.0	0.0	0.0
	Chromophobe renal cell carcinoma	151	118	100.0	0.0	0.0	0.0	0.0
	Oncocytoma	199	147	100.0	0.0	0.0	0.0	0.0
**Tumors of the male genital organs**	Adenocarcinoma of the prostate (primary)	248	232	100.0	0.0	0.0	0.0	0.0
	Adenocarcinoma of the prostate (recurrence)	261	231	100.0	0.0	0.0	0.0	0.0
	Small cell NEC of the prostate	17	16	93.8	6.3	0.0	0.0	6.3
	Seminoma	621	444	0.7	3.2	12.2	84.0	99.3
	Embryonal carcinoma of the testis	50	39	2.6	12.8	20.5	64.1	97.4
	Yolk sac tumor	50	32	25.0	18.8	3.1	53.1	75.0
	Teratoma	50	44	95.5	2.3	2.3	0.0	4.5
	Squamous cell carcinoma of the penis	80	66	98.5	1.5	0.0	0.0	1.5
**Tumors of endocrine organs**	Adenoma of the thyroid gland	114	108	100.0	0.0	0.0	0.0	0.0
	Papillary thyroid carcinoma	392	361	99.4	0.6	0.0	0.0	0.6
	Follicular thyroid carcinoma	158	147	100.0	0.0	0.0	0.0	0.0
	Medullary thyroid carcinoma	107	100	100.0	0.0	0.0	0.0	0.0
	Anaplastic thyroid carcinoma	45	43	100.0	0.0	0.0	0.0	0.0
	Adrenal cortical adenoma	50	44	100.0	0.0	0.0	0.0	0.0
	Adrenal cortical carcinoma	26	26	100.0	0.0	0.0	0.0	0.0
	Phaeochromocytoma	50	50	100.0	0.0	0.0	0.0	0.0
	Appendix, NET	22	12	91.7	8.3	0.0	0.0	8.3
	Colorectum, NET	11	10	100.0	0.0	0.0	0.0	0.0
	Ileum, NET	49	46	100.0	0.0	0.0	0.0	0.0
	Lung, NET	19	17	100.0	0.0	0.0	0.0	0.0
	Pancreas, NET	99	95	98.9	0.0	1.1	0.0	1.1
	Colorectum, NEC	12	10	100.0	0.0	0.0	0.0	0.0
	Gallbladder, NEC	4	4	100.0	0.0	0.0	0.0	0.0
	Pancreas, NEC	15	15	100.0	0.0	0.0	0.0	0.0
**Tumors of hematopoietic and lymphoid tissues**	Hodgkin lymphoma	103	76	100.0	0.0	0.0	0.0	0.0
	Non‐Hodgkin lymphoma	62	54	100.0	0.0	0.0	0.0	0.0
	Small lymphocytic lymphoma, B‐cell type (B‐SLL/B‐CLL)	50	30	100.0	0.0	0.0	0.0	0.0
	DLBCL	114	94	100.0	0.0	0.0	0.0	0.0
	Follicular lymphoma	88	63	100.0	0.0	0.0	0.0	0.0
	T‐cell non‐Hodgkin lymphoma	24	16	100.0	0.0	0.0	0.0	0.0
	Mantle cell lymphoma	18	13	100.0	0.0	0.0	0.0	0.0
	Marginal zone lymphoma	16	10	100.0	0.0	0.0	0.0	0.0
	DLBCL in the testis	16	13	100.0	0.0	0.0	0.0	0.0
	Burkitt lymphoma	5	1	100.0	0.0	0.0	0.0	0.0
**Tumors of soft tissue and bone**	Tenosynovial giant cell tumor	45	44	100.0	0.0	0.0	0.0	0.0
	Granular cell tumor	53	44	100.0	0.0	0.0	0.0	0.0
	Leiomyoma	50	48	100.0	0.0	0.0	0.0	0.0
	Leiomyosarcoma	87	84	100.0	0.0	0.0	0.0	0.0
	Liposarcoma	132	129	100.0	0.0	0.0	0.0	0.0
	Malignant peripheral nerve sheath tumor	13	11	100.0	0.0	0.0	0.0	0.0
	Myofibrosarcoma	26	26	100.0	0.0	0.0	0.0	0.0
	Angiosarcoma	73	66	100.0	0.0	0.0	0.0	0.0
	Angiomyolipoma	91	91	100.0	0.0	0.0	0.0	0.0
	Dermatofibrosarcoma protuberans	21	18	100.0	0.0	0.0	0.0	0.0
	Ganglioneuroma	14	13	100.0	0.0	0.0	0.0	0.0
	Kaposi sarcoma	8	6	100.0	0.0	0.0	0.0	0.0
	Neurofibroma	117	96	100.0	0.0	0.0	0.0	0.0
	Sarcoma, NOS	75	59	98.3	1.7	0.0	0.0	1.7
	Paraganglioma	41	37	100.0	0.0	0.0	0.0	0.0
	Ewing sarcoma	23	18	100.0	0.0	0.0	0.0	0.0
	Rhabdomyosarcoma	7	7	100.0	0.0	0.0	0.0	0.0
	Schwannoma	121	106	100.0	0.0	0.0	0.0	0.0
	Synovial sarcoma	12	11	100.0	0.0	0.0	0.0	0.0
	Osteosarcoma	43	35	100.0	0.0	0.0	0.0	0.0
	Chondrosarcoma	38	17	100.0	0.0	0.0	0.0	0.0

B‐SLL/B‐CLL, B‐cell small lymphocytic/chronic lymphocytic lymphoma; DLBCL, diffuse large B‐cell lymphoma; NEC, neuroendocrine carcinoma; NET, neuroendocrine tumor; NOS, not otherwise specified.

### PLAP expression and histopathological parameters

The relationship between PLAP expression and histopathological data in ovarian, endometrial, and colorectal cancers is summarized in Table 2. The data show that high PLAP expression is linked to advanced pT stage (*p* = 0.0086), nodal metastasis (*p* = 0.0085), and lymphatic (*p* = 0.007) and blood vessel invasion (*p* = 0.0222) in colorectal cancer, while low PLAP expression was found to be associated with advanced pT stage in endometroid carcinoma of the endometrium (*p* = 0.0043). Associations between PLAP expression and tumor phenotype were not found in serous (not otherwise specified) and endometrioid ovarian cancer.

**Table 2:** PLAP immunostaining and cancer phenotype.

		PLAP immunostaining					
		Analyzable (*n*)	Negative (%)	Weak (%)	Moderate (%)	Strong (%)	*P* value
**Colorectal adenocarcinoma**	All cancers	652	89.7	9.2	0.8	0.3	
	pT1	29	100.0	0.0	0.0	0.0	0.0086
	pT2	120	92.5	7.5	0.0	0.0	
	pT3	350	91.4	8.3	0.3	0.0	
	pT4	147	82.3	14.3	2.7	0.7	
	pN−	320	93.8	5.9	0.3	0.0	0.0085
	pN+	324	86.1	12.3	1.2	0.3	
	V0	472	91.9	7.6	0.4	0.0	0.0222
	V+	169	84.6	13.0	1.8	0.6	
	L0	326	94.5	4.9	0.6	0.0	0.0007
	L1	291	84.9	13.7	1.0	0.3	
	Left	571	89.7	9.3	0.7	0.4	0.8544
	Right	76	89.5	9.2	1.3	0.0	
	MMR deficient	21	85.7	9.5	4.8	0.0	0.4772
	MMR proficient	535	90.1	9.0	0.6	0.4	
**Endometroid endometrial carcinoma**	All cancers	173	37.6	31.2	11.0	20.2	
	pT1	114	28.9	30.7	14.9	25.4	0.0043
	pT2	24	45.8	37.5	0.0	16.7	
	pT3‐4	32	56.3	31.3	6.3	6.3	
	pN0	49	32.7	42.9	4.1	20.4	0.1846
	pN+	29	55.2	27.6	6.9	10.3	
**Endometrioid ovarian carcinoma**	All cancers	34	35.3	35.3	5.9	23.5	0.1211
	pT1	24	29.2	37.5	8.3	25.0	0.6409
	pT2	6	66.7	16.7	0.0	16.7	
	pT3	4	25.0	50.0	0.0	25.0	
	pN0	22	40.9	31.8	4.5	22.7	0.7939
	pN1	7	28.6	42.9	0.0	28.6	
**Serous ovarian carcinoma (NOS)**	All cancers	348	49.7	32.8	7.8	9.8	
	pT1	29	41.4	27.6	13.8	17.2	0.6000
	pT2	40	50.0	37.5	5.0	7.5	
	pT3	237	51.9	32.9	7.2	8.0	
	pN0	74	48.6	36.5	4.1	10.8	0.5481
	pN1	153	56.2	30.7	5.9	7.2	

pT, pathological tumor stage; pN, pathological lymph node status; L, lymphatic invasion status; V, blood vessel invasion status; MMR, mismatch repair.

## Discussion

In an immunohistochemical analysis of more than 10,000 tumors analyzed by IHC, it is important to use suitable reagents and protocols. The International Working Group for Antibody Validation (IWGAV) has proposed that antibody validation for IHC on formalin‐fixed tissues should include either a comparison of the findings obtained by two different independent antibodies or a comparison with expression data obtained by another independent method [62]. Here, 76 different normal tissue categories were included in the antibody comparison experiment to ensure that any antibody cross‐reactivity would be detected in our validation experiment. The fact that the antibodies MSVA‐350R and Agilent IR779 both showed strong PLAP staining in chorion and trophoblastic cells of the placenta and weak staining of amnion cells and apical membranes of endocervical, endometrial, and fallopian tube epithelium confirms that these findings are PLAP specific. Most of these results are also consistent with data from three independent RNA screening studies, including the Human Protein Atlas (HPA) RNA‐seq tissue dataset [63], the FANTOM5 project [64, 65], and the Genotype‐Tissue Expression (GTEx) project [5], which also suggest that the uterine cervix is the organ with the second highest PLAP expression following placenta. PLAP RNA expression was not described for endometrium and fallopian tube, but this may be due to the small fraction of the total cells of these organs expressing PLAP. RNAs derived from small structures or rare cell types are largely underrepresented and thus potentially missed in RNA analyses. Lung was also described to produce very limited amounts of PLAP RNA but this is not supported by the findings in our IHC analysis.

It is of note that the strong immunostaining of smooth muscle derived from various organs seen with clone 8A9 was not seen with MSVA‐350R and is thus considered to reflect cross‐reactivity. In line with this interpretation, PLAP RNA expression has previously not been described in smooth muscle cells [5, 64, 65]. Based on these data, the antibody MSVA‐350R was solely used for our tumor tissue analyses. Studies using clone 8A9 have previously described PLAP expression in leiomyoma [49], leiomyosarcoma [49], and angiomyolipoma of the kidney [49, 57]. As we did not find any PLAP staining in a total of 223 tumors of these categories, it appears certain that earlier results were caused by antibody cross‐reactivity and not by true PLAP expression.

The successful analysis of PLAP expression in 12,381 cancers of 131 different tumor types and subtypes confirmed a high frequency of PLAP expression in testicular tumors but also showed that frequent and high‐level PLAP immunostaining occurs in various other tumor types, most commonly derived from the female genital tract, the gastroesophageal, and the pancreaticobiliary system. Our findings observed for seminomas (99%), embryonal carcinoma (97%), and yolk sac tumors (75%) of the testis are largely consistent with the literature [4, 6, 7, 8, 9, 12, 16, 19, 20, 21, 22, 23, 26, 30, 33, 35, 36, 38, 41, 42]. That the highest PLAP positivity rates in extra‐testicular cancers were found in tumors of the female genital tract fits well with the distribution of PLAP expression in normal tissues and also with earlier studies. Several authors have previously described variable levels of PLAP expression in high‐grade serous carcinomas [35, 43, 45, 52, 54, 59], endometroid carcinomas [4, 35, 45, 52], and other variants [4, 35, 52, 58] of ovarian cancer as well as in endometrial cancer [4, 35]. Adenocarcinomas of the stomach and of the esophagus were also among the commonly PLAP‐positive tumors. Previous studies have reported 67% PLAP positivity in a study on 6 adenocarcinomas of the esophagus [4] and in 38% of 8 [4], 23% of 107 [48], and in 0 of 2 gastric adenocarcinomas [35]. Moreover, the TCGA database described elevated PLAP expression in 60% of 354 gastric adenocarcinomas [1].

From a diagnostic point of view, it is important to keep in mind that very high PLAP expression levels, which are often considered characteristic for germ cell tumors, predominated in germ cell tumors but also occurred in multiple additional tumor entities. These included – in addition to those mentioned above – further clinically important and frequent cancer types such as adenocarcinoma of the lung, urothelial cancer, colorectal adenocarcinoma as well as mucoepidermoid carcinoma of salivary glands. It is also noteworthy that weak PLAP expression limited to a small subset of tumor cells can occur in a wide variety of tumor entities and must not be viewed as a strong argument for the germ cell origin of a cancer. It was not within the scope of our study to analyze molecular mechanisms and functional consequences of PLAP expression in these cancers. However, the availability of clinicopathological data for some of the tumor entities that expressed PLAP in a significant fraction of cases enabled an analysis of the potential clinical significance of PLAP expression. Finding a link between PLAP upregulation and colon cancer aggressiveness supports the concept of targeting PLAP in colon cancers [66]. That the respective findings were inverse between endometrial and colorectal cancer might suggest that the tumor biologic role of PLAP expression can vary between tumor entities.

**Figure 4:**
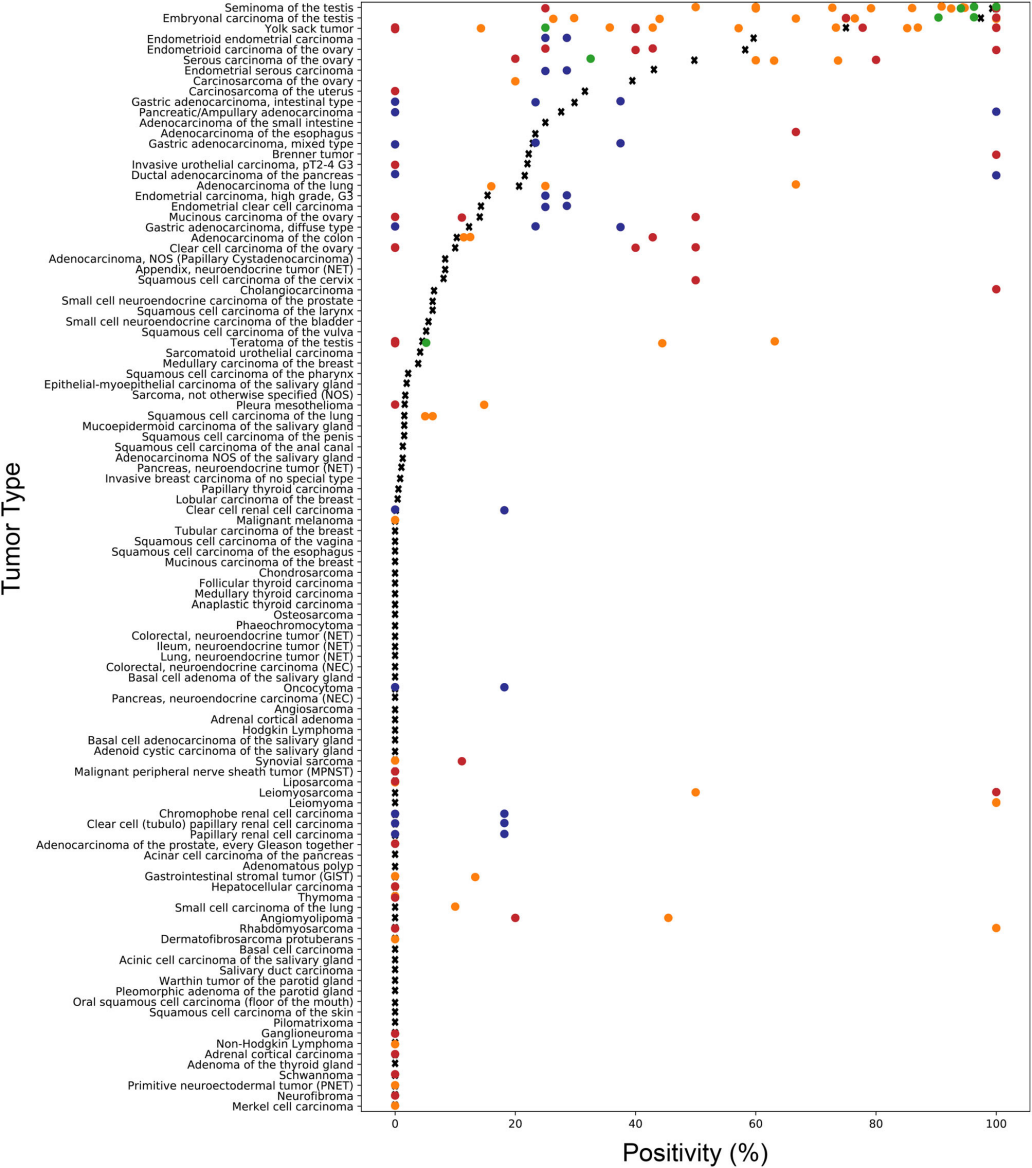
Graphical comparison of PLAP data from this study (x) in comparison with the previous literature (dots). Red: *n* = 1–9, orange: *n* = 10–50, green: *n* > 51. For comparison purposes, studies that did not differentiate between different tumor subtypes were marked with blue dots and the overall positivity rate was applied to the different tumor subtypes present in our tumor microarrays. All studies are referred to in the reference list.

The data from this study provide a comprehensive ranking list of tumors according to their PLAP expression across a large variety of tumor entities. It is a strongpoint of our study that all tissues were stained in 1 day under exactly the same experimental conditions and that one expert pathologist interpreted all immunostains, resulting in as much standardization as possible. It is almost certain that the use of different protocols, antibodies, interpretation criteria, and thresholds used to define ‘positivity’ have jointly caused the high diversity of literature data on PLAP expression in cancer (summarized in Figure 4). The frequencies described in this study are thus specific to the reagents and protocols used in our laboratory. It is expected that different experimental conditions would have changed the PLAP positivity rates – especially in the cancers with low expression levels – but would have little impact on the tumor ranking based on the PLAP positivity rates.

In summary, our data show that PLAP can be highly expressed in a variety of tumor types. Besides germ cell tumors, which show the highest PLAP expression prevalence, high‐level PLAP expression can be found in cancers from the female genital tract, the gastroesophageal, and the pancreaticobiliary system as well as in a few other tumor types. Low‐level PLAP expression can be found in various other tumor entities and should generally not be viewed as a strong argument for germ cell neoplasia.

## Author contributions statement

VR, TK, RS and GS designed the study. VR, NG, AML, EB, AM, CW, SW, CF, KM, PL, RU, WW, FJ, SM, CB, AM, SS and TK performed the immunohistochemical analyses and/or contributed to the pathological validation of the tumors, the TMA construction, and data collection. MK, CH‐M and RS carried out the data analyses. GS, RS, TK and VR wrote the first draft of the manuscript. All authors contributed toward data analysis, drafting and critically revising the paper, gave final approval of the version to the published, and agree to be accountable for all aspects of the work.
